# Compensatory hallucinogenesis across three neuropsychiatric disorders: a Bayesian account

**DOI:** 10.1093/braincomms/fcag001

**Published:** 2026-01-07

**Authors:** Raina Vin, Jordan Galbraith, Rashina Seabury, Hae Young Yi, Gabriela Hernández-Busot, Lucas Oland, Boris Epie, Anne Trainer, Carolyn Fredericks, Albert R Powers

**Affiliations:** Yale School of Medicine, Yale University, New Haven, CT 06510, USA; Yale School of Medicine, Yale University, New Haven, CT 06510, USA; Yale School of Medicine, Yale University, New Haven, CT 06510, USA; Yale School of Medicine, Yale University, New Haven, CT 06510, USA; Yale School of Medicine, Yale University, New Haven, CT 06510, USA; Yale School of Medicine, Yale University, New Haven, CT 06510, USA; Yale School of Medicine, Yale University, New Haven, CT 06510, USA; Yale School of Medicine, Yale University, New Haven, CT 06510, USA; Yale School of Medicine, Yale University, New Haven, CT 06510, USA; Yale School of Medicine, Yale University, New Haven, CT 06510, USA

**Keywords:** Charles Bonnet syndrome, dementia with Lewy Bodies, psychosis, sensory signal disruption, Bayesian computational framework

## Abstract

Emerging evidence suggests that hallucinations may arise because of an over-reliance on prior knowledge during perception. While best established in psychosis-spectrum illness, data also support the presence of this abnormality in other hallucination-prone neuropsychiatric illnesses that vary in their association with disruption of sensory circuits. In this piece, we ask whether an over-weighting of expectations may be conceived of as a compensatory response to degraded incoming sensory information.

We make the case that visual hallucinogenesis across a wide array of neuropsychiatric disorders can be captured within a common Bayesian computational framework, as a compensatory response to sensory signal disruptions at different levels of the visual processing hierarchy. We focus on three specific disorders (Charles Bonnet syndrome, dementia with Lewy Bodies and psychosis) with prominent visual hallucinations and highlight the fact that these disorders describe a spectrum of visual impairment where the overtness and localization of the visual processing disruption is reflected in the characteristics of the emergent visual hallucinations. We examine how discrete sensory disruptions in Charles Bonnet syndrome translate to hallucinations via known circuits, and then how different disruptions in dementia with Lewy Bodies and Schizophrenia may lead to hallucinations with distinct phenomenology, comorbidities and circuit involvement. Finally, we appeal to emerging computational theories to unite these observations under a common conceptual umbrella.

Taken together, this work presents a means of understanding how sensory disruptions could interact with other aspects of cognitive and neural architecture to produce hallucinations across neuropsychiatric disease. It is our hope that this framework will help in efforts to identify pathophysiologically distinct patient subgroups and new pharmacological and circuit-based interventions.

## Teaser

In this review, Vin *et al*. propose a unified Bayesian framework for visual hallucinations across three neuropsychiatric disorders: Charles Bonnet syndrome, dementia with Lewy bodies and psychosis. They suggest hallucinations arise as a compensatory response to different levels of sensory signal disruption.

## Graphical Abstract

**Graphical Abstract:**
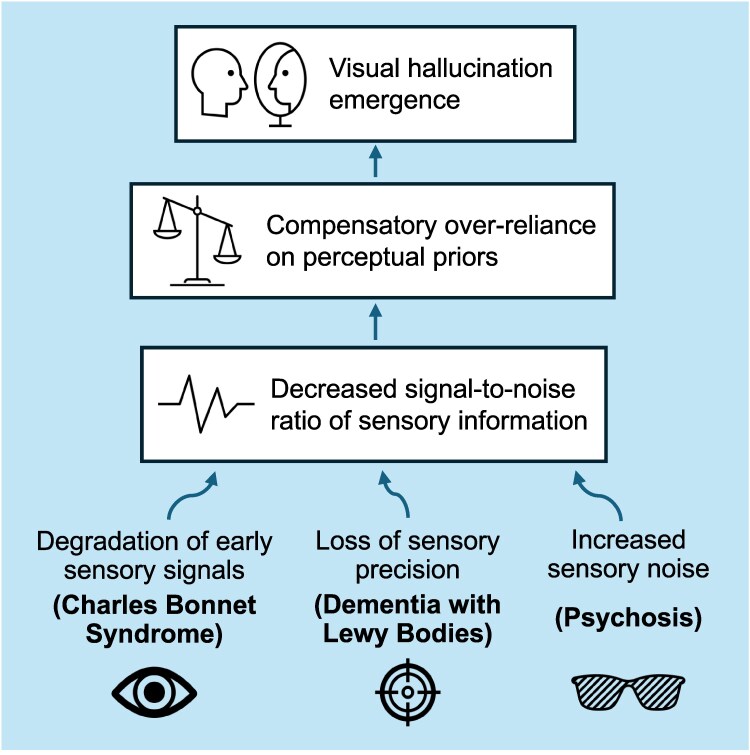

## Introduction

Human perceptual systems make an extremely difficult task look automatic. Charged with inferring the contents of the environment on a moment-to-moment basis, perceptual systems perform something of a magic trick, convincing us that their outputs are the unadulterated truth about what is out there. For this reason, it is tempting to view perceptual processing as a passive, deterministic reception of information. However, decades of research in systems and computational neuroscience now offer a compelling alternative account: perception is the conclusion of an inferential process in which existing beliefs about the causes of sensory information are combined with new evidence in a procedure approximated by Bayesian statistics.^1,2^ This framework has proven useful in interpreting the results of perceptual experiments ranging from the use of lip-reading cues^3^ and sentence context^4^ in understanding speech in noisy environments, to the use of shading to infer three-dimensional surfaces in visual art.^5^

Beyond its usefulness in normative contexts, the Bayesian computational framework has helped to identify the theoretical underpinnings of aberrant perception, including hallucinations.^6-8^ Across a range of disorders and general-population samples, evidence supports the possibility that hallucinations may arise from an over-reliance on perceptual beliefs over incoming sensory evidence, driving the conclusion that the environment contains something it does not.^8-15^ Although these insights are important for understanding the brain states leading directly to hallucination emergence,^10^ no work has yet fully explored why an overreliance on perceptual beliefs might arise in the first place—whether via a primary mechanism likely driven by alterations in neuromodulators like dopamine (DA)^13,15^ or by secondary mechanisms in which sensory disruptions might lead to a compensatory overreliance on perceptual beliefs.^6,16^

An examination of the hallucinations that arise within each of the constituent sensory systems may allow us to determine if there is any support for the secondary account of hallucinogenesis. Deafferentation hallucinations, arising after some insult to the sensory systems,^17^ appear to be driven by a conserved mechanism across sensory modalities. Starting with the recognition of visual disruptions leading to Charles Bonnet syndrome (CBS) in 1760,^18^ hallucinations have been documented in all modalities following deafferentation: musical ear syndrome^19^ and tinnitus^20^ in audition, phantom limb syndrome^21^ in somatosensation, gustatory and olfactory hallucinations arising after sudden loss of sensation^22^ and movement hallucinations after vestibular injury.^23^ Beyond these disease processes, sensory deprivation is known to reliably produce hallucinations across sensory modalities.^24^ Although these examples make clear that deafferentation is one possible pathway towards hallucinogenesis, it is unclear how representative this motif is across neuropsychiatric disease, even in cases where an obvious insult does not precede hallucination onset.

**Figure 1:**
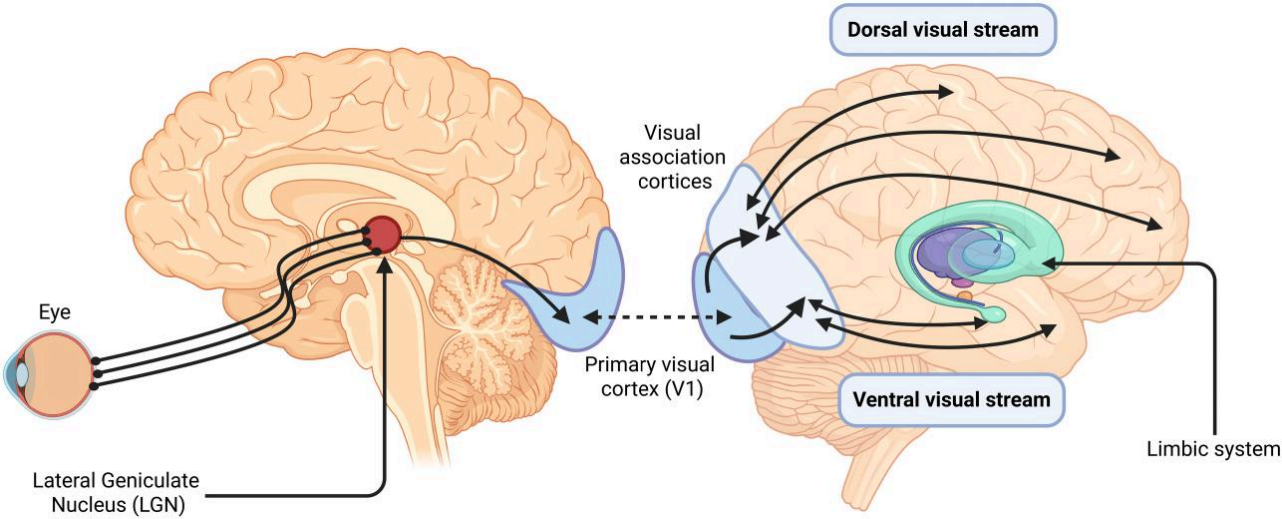
Overall organization of the visual circuit. Visual information detected by the eye is transmitted via the optic nerve to the LGN of the thalamus. From the LGN, signals project to the primary visual cortex (V1) and subsequently to higher-order visual association cortices. Processing then diverges into two major pathways: the dorsal visual stream, which supports spatial processing and visually-guided action (‘where’ pathway), and the ventral visual stream, which mediates object identification and recognition (‘what’ pathway). The ventral stream also projects to the limbic system, linking visual information with affective and mnemonic processing. Arrows indicate the principal projection pathways of the visual circuit. Created in BioRender. Vin, R. (2025) https://BioRender.com/zrn6pkt.

In this piece, we choose to focus on visual hallucinations (VH) arising within three different neuropsychiatric disorders. Beginning with the disorder with the clearest tie to deafferentation (CBS), we move to two others with progressively less obvious links to sensory dysfunction [dementia with Lewy bodies (DLB] and psychosis). Our goal is to use the visual system as an anchor (Fig. 1), drawing distinctions and parallels across the three disorders and examining insults to this system that may degrade signal integrity, thereby contributing to the emergence of hallucinations.

To ground this discussion, we conducted targeted literature searches in PubMed and Google Scholar using combinations of the key disorder names—‘Charles Bonnet Syndrome’, ‘Dementia with Lewy Bodies’, and ‘Psychosis’—with terms such as ‘visual hallucinations’, ‘visual processing’, ‘neurotransmitter dynamics’, ‘visual system pathology’, ‘connectivity’, and ‘brain atrophy’. We prioritized peer-reviewed empirical studies that presented clear, interpretable findings relevant to the mechanisms of visual hallucinations. Among several theoretical works on visual hallucinogenesis is an excellent recent consensus review by Collerton *et al*. (2023)^25^ that explores a broad range of explanatory models. In contrast, our goal is to specifically evaluate the plausibility of the Bayesian account of visual hallucinogenesis by synthesizing converging lines of evidence within these three disorders. By systematically comparing CBS, DLB and psychosis, we hope to survey a representative range of identifiable sensory disruptions that might underlie hallucinations within a single, unifying mechanistic framework, while also accounting for shared phenomenological features, comorbidities and response to existing treatments. We intend for this approach to offer novel insights into the clinical heterogeneity of hallucinations and suggest new directions for targeted intervention.

## Charles Bonnet syndrome

### Prevalence and phenomenology of visual hallucinations

CBS is characterized by VH arising secondary to loss of vision or visual acuity. It is caused by a direct lesion to the primary visual pathway, ranging from intrinsic eye pathology^26^ to damage of the optic nerve, optic radiations or occipital cortex.^27,28^ Patients typically maintain insight into the unreality of VH and most find them tolerable,^29,30^ though 32% report emotional distress.^31^

While CBS can occur in individuals at any age and has been observed in children,^32^ it is more prevalent among the elderly^33-35^ and is often associated with age-related eye disorders, such as macular degeneration, diabetic retinopathy, cerebral infarctions, and glaucoma.^26,36^ The prevalence of CBS varies from 10% to 38% in individuals with vision loss,^37-45^ due to heterogeneity in diagnosis across ocular disorders. Patient reluctance to disclose symptoms and lack of awareness of CBS may further contribute to this variability.^29,46,47^

The emergence of VH is typically slower in individuals with progressive vision loss, often taking more than a year, compared to acute cases where VH may appear within hours to days.^26,35^ Three distinct patterns of the course of CBS have been identified: episodic (a short phase of hallucinations lasting days to months that resolves without intervention), periodic (alternating phases of hallucinations and remission), and continuous (persistent hallucinations without remission).^35^ Across these courses, VH often begin as simple percepts and may gradually progress to more complex images over time. Simple hallucinations often include geometric patterns or basic shapes, which may appear in black and white or, more commonly, in vivid color.^35^ Complex hallucinations in CBS involve more detailed and dynamic imagery, such as animals, human figures, faces, buildings or scenery.^31,33,48,49^ These images can vary in size, appearing miniaturized, normal-sized, or enlarged (‘larger than life’) and are perceived as part of the external environment, standing out in sharp contrast to the blurred perception of real objects in individuals with impaired vision.^35^

Typical CBS hallucinations are limited to the realm of vision and do not include components from other sensory modalities. They occur while the individual is alert with eyes open, featuring a sharply focused image that appears suddenly without any trigger or voluntary control. These hallucinations are usually short in duration, lasting from seconds to minutes on average and subsequently disappear as abruptly as they began.^31,49,50^ Although they are often static, hallucinations with movement have been observed as well.^51^ Images may shift within the visual field but remain unanimated, or may be animated and characterized by rhythmic, repetitive actions, such as walking, head-nodding, or marching in procession.^51^ These episodes can vary in frequency, occurring from once a month to several times a day.^31,49,50^ Atypical hallucinations have also been documented in CBS, which may incorporate elements of the individual's psychological state,^52^ resembling dream-like experiences rather than the sharply focused, externalized images typical of CBS.

### Visual system pathology

#### Functional disruptions in vision-related regions

Early theories of CBS focused on the hypothesis that the VH result from deafferentation of incoming sensory inputs to the occipital lobe, resulting in aberrant hyperexcitability of early cortical visual regions.^53-56^ Supporting this view, individuals with CBS demonstrate elevated baseline activation of visual cortex and relatively reduced activation during processing of external visual stimuli.^56,57^ They also show higher steady-state visual evoked potentials in early visual cortical regions in response to distractor checkerboard stimuli during a visual target-monitoring task, compared with participants with macular degeneration without CBS, and age-matched healthy controls.^58^

In contrast, structural investigations of CBS remain limited. Although some studies of CBS patients and individuals with eye disease but no VH have reported reduced occipital grey matter volume and altered white matter microstructure—marked by decreased fractional anisotropy and increased diffusivity in the corpus callosum, occipital tracts and anterior thalamus—across both groups,^27^ direct comparisons between these groups remain limited and show no significant differences in DTI or VBM measures. The paucity of evidence underscores the need for further research into the role of white matter integrity in CBS.^27,59^

### Implications for treatment

The mainstay of therapy for the VH of CBS is treatment of the underlying visual disorder (where possible), with most other strategies relying on sparse evidence.^60,61^ In individuals with reversible conditions, such as cataracts, VH typically subside naturally after vision is restored.^29^ VH may also terminate spontaneously as the brain adapts to sensory deficits.^35^ Limited awareness of CBS and its underlying neural mechanisms poses a significant challenge to developing effective, more advanced treatments.

#### Direct modulation of cortical activity

With growing insights into the role of visual cortical excitability in CBS, novel interventions targeting this mechanism are being explored as potential treatments. A recent randomized placebo-controlled crossover trial utilizing inhibitory transcranial direct current stimulation to visual cortex in sixteen individuals with CBS found a significant reduction in frequency of VH (Cohen’s *f* = 0.75) as measured by the North East Visual Hallucinations Interview.^62^ Consistent with the idea that VH in CBS are driven by visual hyperexcitability in the absence of modulatory visual sensory input, individuals with greater occipital excitability during a baseline EEG reported a more positive treatment response than those with lower baseline excitability. More work on the role of cortical inhibition and its relationship with bottom-up noise is needed to further parse this relationship.

#### Modulation of cholinergic signalling

There is no standardized pharmacological treatment for CBS, though medications targeting serotonergic or dopaminergic pathways are occasionally utilized.^60,63,64^ A heightened focus on predictive processing in theories of VH emergence has shifted attention to the possible involvement of acetylcholine in VH pathogenesis, with potential implications for CBS.^6,65,66^ A growing body of evidence suggests that cholinergic tone increases the weighting of incoming sensory information^67-69^ and modulates the weight afforded to prediction errors.^70^ To our knowledge, only one direct study involving CBS patients (*n* = 2) exists that investigates both efficacy of cholinergic manipulation as a treatment for CBS-associated VH and the neural correlates of these effects. Hanoglu and colleagues^71^ found that administration of rivastigmine in combination with alpha-lipoic acid resulted in a reduction of hallucinations in two patients with CBS. This finding, in addition to several case studies,^36,72,73^ suggests that targeting cholinergic signalling may offer therapeutic potential for CBS, though further research is needed to clarify this relationship.

It has been noted that CBS-related VH improve when there is a total lack of visual input (i.e. when eyes are closed), which has been theorized to signify an increase in certainty of bottom-up signal being zero, leading to this reduction in VH.^74,75^ Most individuals with ocular disorders and low vision do not experience VH, raising questions of whether CBS indicates a failure of the brain to mount an appropriate compensatory cholinergic response to the insult, leading both to hyperexcitability of early visual cortical regions and to VH.

### Charles Bonnet syndrome in a Bayesian framework

In a Bayesian framework, perception is the result of the brain making inferences about the world based on prior beliefs and sensory evidence.^76^ In normal vision, the brain integrates robust visual input with prior beliefs to produce accurate perceptions about the environment. However, in CBS, the degradation of incoming visuosensory information causes the visual system to rely more heavily on priors to fill in the gaps, ultimately driving the emergence of VH. This is broadly consistent with older theories of CBS emergence by deafferentation,^55,77^ i.e. that visual cortical hyperactivity (and thus VH) result from the absence of reliable input. While this spontaneous early visual cortical activity from neural disinhibition could account for the simple VH of geometric patterns or shapes observed in CBS—akin to visual auras in migraine or seizures-related phenomena^78-81^—the more complex and elaborate visual hallucinations often reported in CBS^31,33,48,49^ may be more consistent with top-down modulatory effects on hyperactive visual cortex.

**Figure 2:**
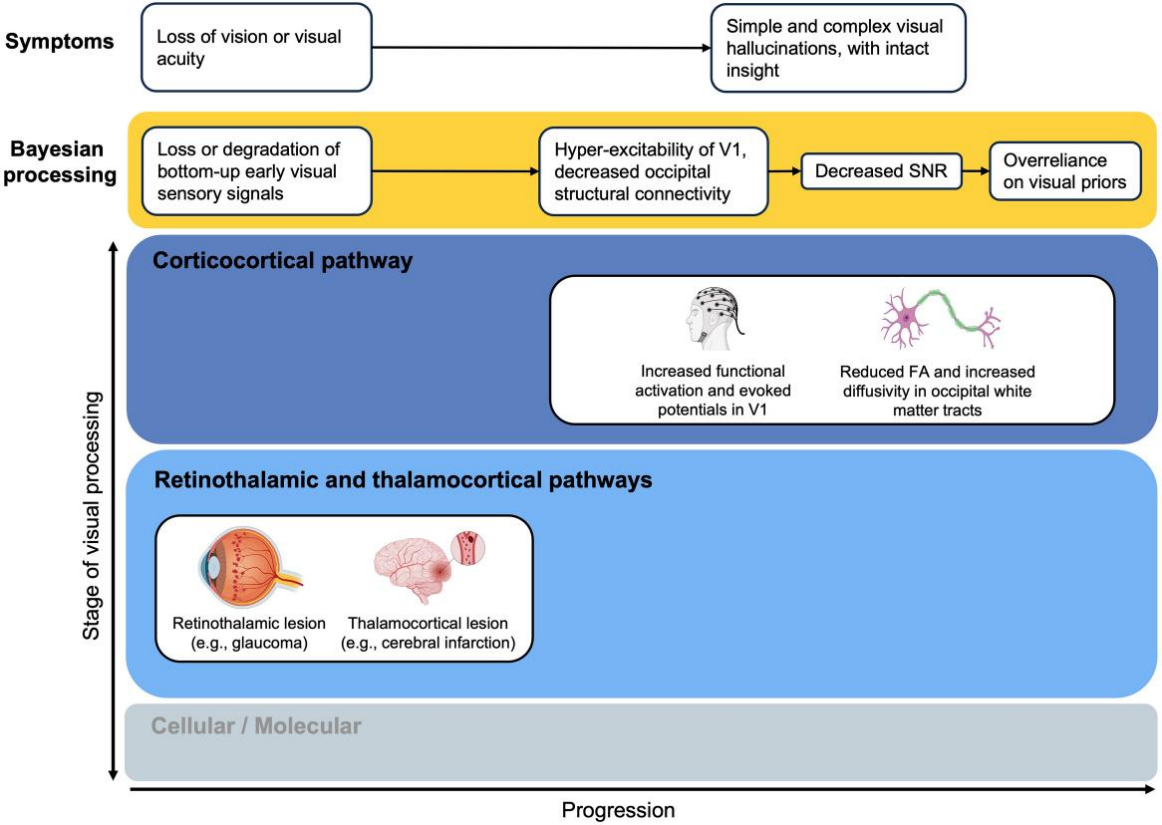
Charles Bonnet Syndrome within a Bayesian framework. The x-axis depicts syndrome progression, and the y-axis illustrates successive stages of visual processing, from cellular/molecular mechanisms to retinothalamic and thalamocortical pathways, and finally corticocortical circuits. Boxes surrounded by yellow mark the aspect of Bayesian processing disrupted at each stage, with associated symptoms shown directly above. In the early phase, lesions in the retina or thalamus degrade bottom-up visual signals, leading to reduced visual acuity. Concurrently, functional alterations such as increased V1 activation and structural changes including reduced fractional anisotropy and elevated diffusivity in occipital white matter tracts emerge. These alterations result in diminished signal-to-noise ratio, promoting a compensatory over-reliance on visual priors. SNR: signal-to-noise ratio; FA: fractional anisotropy. Created in BioRender. Vin, R. (2025) https://BioRender.com/sxoywz6.

The restriction of CBS-associated pathologies to the earliest stages of the visual pathway^26,36,49^ may explain the relative sparing of higher-order cortical regions implicated in reality monitoring processes that distinguish between internally and externally derived information,^82,83^ and thus preserve insight among those with CBS. Neuroimaging studies of CBS patients predominantly find abnormalities in primary visual processing regions and association cortices, with VH appearance coinciding with transient activation of these regions.^56,84^ The Bayesian framework supports the possibility that VH emerge when priors are weighted more heavily than sensory information, especially in conditions where that information is lacking (Fig. 2).

## Dementia with Lewy bodies

### Prevalence and phenomenology of visual hallucinations

DLB is a progressive neurodegenerative disease that accounts for up to 24.4% of all dementia cases^85^ and is associated with the accumulation of alpha-synuclein deposits, also known as Lewy bodies (LB).^86,87^ DLB is formally distinguished from Parkinson’s disease with dementia (PDD) on the basis of timing, with the latter defined by a motor syndrome that arises more than a year prior to the cognitive syndrome. At least to some extent, DLB and PDD likely exist on a spectrum, and many studies include mixed diagnostic samples, limiting our ability to speak to findings known to be specific to DLB.

That said, some clinical features starkly distinguish DLB from other alpha-synucleinopathies and from typical presentations of PDD. In addition to dementia, which typically involves deficits in visuospatial and executive function disproportionate to milder memory symptoms, core criteria of illness include early, well-formed, vivid VH, fluctuating cognition, a mild and typically symmetric parkinsonism, and REM sleep behaviour disorder (RBD), the last of which is often a prodromal state that can precede other symptoms by more than 10 years.^87,88^ VH are nearly universal in DLB, with up to 90% of patients experiencing them during the course of illness.^87,89-93^ In contrast, psychotic symptoms in Parkinson’s disease typically emerge later in the disease course, if they occur, and are often confounded by pharmacological treatments.^94,95^

Our decision to examine DLB, rather than Parkinson’s disease psychosis, is deliberate. The study of early-emerging VH in DLB provides a unique opportunity to isolate the initial circuit dysfunctions and pathophysiological changes that drive the onset of psychosis, offering clearer mechanistic insights than can be obtained from Parkinson’s disease psychosis. This distinction is particularly important given the long-standing conflation of DLB and PDD, despite important clinical and pathological differences between the conditions.

VH in DLB most often take on complex, animated forms, including familiar and unfamiliar adults or children, deceased family members and small animals.^95,96^ The frequency of VH ranges from monthly to continuous and duration from seconds to hours.^92,96^ While complex VH are typically the first subcategory of true hallucinatory symptoms to emerge in DLB,^97^ many individuals also experience abnormal visual perceptual phenomena (sometimes referred to as ‘minor VH’). These include presence phenomena,^98^ passage phenomena^96,99^ and illusions^100^ that may precede or accompany complex VH. Like people with CBS, DLB patients classically have insight into their VH and are not distressed by them, particularly during the early stages of illness,^101-103^ though this often changes with disease progression and cognitive impairment.^87,95,100,104,105^

While VH are far more common, auditory and tactile hallucinations have been reported in variable percentages of patients with DLB.^97^ Unlike in psychosis, auditory hallucinations (AH) are relatively rare, with a general prevalence of 30.8%; the prevalence of patients with AH and without VH is 0.6–15%.^89,106-108^ Most often, AH occur later and bind to recurrent VH to create fused multimodal hallucinations that increase in prevalence in later stages of illness.^89,106,109-112^ AH typically consist of human voices and are described as the ‘soundtrack’ of the accompanying VH.^106^

A secondary feature of DLB is systematized delusions, with several clinical and neuropathological DLB cohorts reporting a prevalence of around 50%.^109,113,114^ In contrast to patients with psychosis, most DLB patients with delusions experience VH first, with delusions typically arising 2–3 years into the course of illness.^115-118^ Notably, most delusions of DLB can be thought of as entrenched misperceptions, with reduplicative paramnesias—a class of delusions marked by the belief that someone or something has been replaced by an identical-appearing imposter—being the most common.^112,119^ Capgras syndrome, a reduplicative paramnesia for a specific person such as a loved one, is the classic delusion associated with DLB and nearly always co-occurs with VH, pointing to a common etiology.^95,114,117,118,120^

### Comorbid visual processing deficits

Visual symptoms are present very early in the course of DLB. Individuals with RBD, more than 90% of whom will convert to clinical alpha-synucleinopathy (typically DLB or PD) within 14 years,^121,122^ display subclinical impairments in visuoperception, visuospatial construction and colour vision starting 10–16 years prior to conversion,^88,100,123-127^ and these predict progression to DLB over PD.^88,128^ Greater impairment in visuospatial tasks that utilize the dorsal visual stream differentiates early DLB from other forms of dementia.^129,130^

The visual attention deficits seen in DLB could also be a key driver of VH.^87,131^ In visual search tasks, patients with DLB exhibit poor attentional guidance compared to Alzheimer’s disease (AD), as well as greater eye movement alterations.^92,132,133^ These findings accompany greater impairments in attention testing,^134,135^ as well as substantial changes in structural and functional connectivity among key networks related to visual attention in patients with DLB with VH versus without them.^136-138^ In mixed samples of DLB and PDD, patients show decreased visual exploration of naturalistic complex scenes as well as increased centre bias compared to AD and healthy controls,^139^ which correlates with disease severity and could relate to the tendency of early visual misperceptions, such as illusions and passage phenomena, to occur in the visual periphery.^96,99,100^

### Visual system pathology

#### Lewy body pathology

Lewy bodies, the hallmark neurodegenerative pathology associated with DLB, are aggregated alpha-synuclein plaques that follow a stereotypical progression from the olfactory bulbs to the brainstem and/or limbic regions and finally the neocortex.^140-143^ Their neurotoxicity stems from disruption of the mitochondria, endoplasmic reticulum, synapse and nucleus, as well as the activation of apoptosis cascades.^144^ The vast majority of alpha-synuclein, however, exists in the form of much smaller aggregates located at presynaptic terminals, which are also increasingly recognized as a major cause of neurodegeneration.^145^

While early staging systems were tailored for individual alpha-synucleinopathies, the more recent Unified Staging System for Lewy Body Disorders captures spread across syndromes,^140,141^ noting that early limbic-predominant pathology tends to predict a DLB syndrome while brainstem-predominant pathology is more associated with PD.^140^ Calabresi and colleagues^146^ describe a severe phenotype of alpha-synucleinopathy that includes earlier involvement of limbic and prefrontal areas and is associated with more severe cognitive and psychiatric symptoms, including VH specifically. Notably, the density of Lewy bodies in the temporal lobes (including amygdala) strongly predicts the presence of VH,^147^ and the amygdala in particular appears to be a seed for prion-like spread of alpha-synuclein to other cortical regions in DLB^148^ including the anterior insula, which we and others have implicated in hallucinations.^6,9^

Lewy body pathology also disrupts the visuo-amygdaloid pathway more generally. While the greatest Lewy body burden is in the amygdala itself, patients both with and without VH have significant pathology in secondary visual pathways including the pulvinar and V2-V5, but much more limited Lewy body burden in the lateral geniculate nucleus (LGN) and primary visual cortex (V1),^149^ consistent with a greater role of visual attention deficits than deficits in incoming sensory percepts in hallucination generation.^150-152^ That the amygdala is particularly implicated suggests a potential deficit in incorporating emotional and cognitive states. This would implicate interruption of both top-down contextual visual processing as well as bottom-up sensory-state weighting in VH development in DLB.

#### Morphological and functional abnormalities of the retina

Optical coherence tomography (OCT), a non-invasive imaging technique, shows thinning of the retinal nerve fibre layer^153-155^—the bundle of retinal ganglion cell axons that transmits visual signals from the retina to the visual cortex through the optic nerve—along with reduced macular^153,156,157^ and granular cell layer-inner plexiform layer^153,154,156^ thickness in DLB compared with healthy controls. Angiography studies (OCTA) further demonstrate alterations in vessel density and perfusion compared to healthy individuals.^153,154,158^ These retinal alterations are clinically significant, correlating with measures of memory, cognition, and hippocampal atrophy.^155,156^

#### Structural disruptions in vision-related regions

Early degeneration of the nucleus basalis of Meynert (NBM) disrupts white matter tracts to the cortex and amygdala in DLB.^159-162^ Reduced fractional anisotropy and increased mean, radial, and axial diffusivity in these pathways have been linked to impairments in global cognition, visuospatial function, attention, executive function, and episodic memory^160,163-165^ as well as altered functional connectivity of visual networks, the default mode network (DMN), and the ventral attention network (VAN) in patients with VH.^136^ Moreover, disruptions in these early visual pathways appear to precede development of VH: in one study, radiologists could differentiate between individuals with and without VH among a mixed sample of patients with PD, PDD or DLB based on the structure of their optic radiations. A longitudinal analysis of the patients initially without VH revealed that hallucinations emerged at follow-up only in those with abnormal optic radiation structure at baseline.^166^ Abnormalities in the longitudinal fasciculi have also been associated with VH presence and severity,^163,165^ though findings vary across studies.^167^ Reports of greater global white matter hyperintensity burden in DLB show similar variability. The occipital and posterior periventricular regions are most often implicated, with associations reported to dementia severity, neuropsychiatric symptoms and cognitive measures, although evidence for links to VH is inconsistent.^168-173^ Overall, findings on white matter alterations in DLB are highly divergent, and it remains uncertain whether they reflect disease-specific mechanisms, shared neurodegenerative processes, or nonspecific ageing-related changes.^174-177^ Longitudinal studies beginning in prodromal cohorts are needed to better characterize white matter changes specific to DLB and their contribution to VH emergence.

#### Functional disruptions in vision-related regions

Changes in functional brain circuitry contribute to VH in DLB. Connections between the visual network, dorsal attention network (DAN), VAN, and the DMN are particularly implicated.^137,138,165,178,179^ Complex VH have been associated with poorer functional coupling between bottom-up and top-down visual streams, with decreased functional connectivity between visual networks and key regions in the DMN, as well as between visual networks and the VAN.^74,136^ Reductions in DAN activation are of equal or greater prominence than deficits in primary and association visual circuitry,^180-184^ and disrupted DAN function may increase reliance on the DMN and VAN in processing ambiguous stimuli.^182,183,185,186^

Hallucinations in DLB often begin with minor disruptions like illusions and passage/presence phenomena. Interestingly, these have been associated with decreased functional connectivity between early visual areas and ventral visual streams, as well as decreased connectivity between early visual areas and the brainstem.^96^ These data hint at the possibility of bottom-up functional changes underlying minor, mostly early visual disruptions and altered connectivity between top-down and bottom-up circuits implicated in complex VH. This bias towards early sensory disruptions in the initial stages of DLB could potentially lay the groundwork for insightful VH, which deteriorates as higher-order cortical regions become involved in later stages of the illness.

### Implications for treatment

#### Modulation of cholinergic signalling

NBM degeneration in DLB disrupts diffuse cholinergic projections to the occipital lobe, optic radiations and temporal lobe,^187,188^ as well as deeper structures including epithalamus and insula.^189^ From a Bayesian perspective, acetylcholine works in tandem with the dopaminergic system as an uncertainty encoder that modulates weighting of incoming information to shift attention.^190,191^ Thus, early cholinergic deficits in visual processing areas in DLB might result in a relative over-weighting of top-down information due to decreased weighting of bottom-up information.^8,192-195^

Acetylcholinesterase inhibitors (AChEIs), which prevent acetylcholine breakdown and enhance its synaptic levels, offer significant benefits for treating VH in DLB,^196-199^ with 60% of patients experiencing a 30% improvement in VH in one large trial of rivastigmine.^200^ Consistent with observations that acetylcholine modulates the weighting of incoming sensory information, AChEIs may also treat the visuospatial symptoms of DLB,^196-198^ while their withdrawal can worsen visual attention.^201^

#### Modulation of dopaminergic signalling

Alpha-synuclein pathology in key dopaminergic nodes leads to a widespread dysregulation of dopaminergic signalling.^202^ In a post-mortem study, DLB patients exhibited reduced D2 binding in the putamen and reduced DA concentration in the caudate, with no compensatory increases in dopaminergic turnover.^203,204^  *In vivo* studies using DA transporter imaging have further found an inverse relationship between striatal and substantia nigra DA transporter levels and frequency and severity of VH in DLB.^205,206^ This is consistent with a role for the DA transporter in maintaining stable extracellular DA levels that, when disrupted, could result in DA dysregulation and transient overstimulation. These in turn might contribute to the fluctuations in cognition and VH seen in DLB.^207-209^ Other groups have instead posited that cholinergic defects are the primary driver of symptomatology and compromise dopaminergic neurotransmission as a secondary effect.^210^ Still others suggest that the effect of reduced caudate DA on visuospatial and cognitive function in DLB is mediated by hypometabolism in parietal and occipital regions.^211^

Studies of antipsychotics in DLB are limited, given that many patients respond poorly; severe sensitivity to neuroleptics is a supportive feature for the diagnosis of DLB.^87^ Quetiapine is considered the antipsychotic of choice despite the potential of motor and psychiatric side effects, due to its relatively lower risk of inducing neuroleptic sensitivity.^87,212-215^ A novel inverse 5-HT2A receptor agonist, pimavanserin, has shown some promise in addressing VH in DLB with lower risk of worsening parkinsonism given its lack of dopaminergic effects, although no large randomized controlled trials in DLB exist.^216-219^

DA agonists, too, are used with caution in DLB patients, though more commonly, for management of parkinsonian symptoms. In this context, they are effective for parkinsonism in only about one third of patients and are associated with a high risk (also about one in three) of worsened VH and other psychiatric symptoms.^220-222^ In a study of 19 DLB patients who received pro-dopaminergic medications, for example, only four had some motor benefit without worsening of VH.^220^

#### Non-pharmacological interventions

Behavioural strategies to decrease sensory noise and focus visual attention away from VH-associated aspects of the visual scene^223,224^ have proven helpful in reducing the VH of DLB in small studies. Similarly, reducing social isolation in order to improve low arousal and attention may be of benefit,^87^ reminiscent of the role of social deafferentation in the hallucinations of psychosis.^225^

### DLB in a Bayesian framework

While DLB is associated with early-emerging abnormalities in visual processing as early as the retina or optic radiations, its most profound impact in patients with complex VH is on downstream aspects of visuospatial processing, and particularly visual attention. If the primary driver of bottom-up signal degradation in DLB is low weighting of visuosensory information, rather than disrupted visual input, boosting cholinergic tone in this population should normalize weighting of visuosensory inputs and decrease VH frequency. This appears to be the case: AChEIs improve VH in a majority of patients and improve visual attention and visuospatial test scores.^196,197^ As our model would predict, they also reduce the tendency of DLB patients to perceive meaningful images such as faces in noise.^198^ Early illusions and presence/passage phenomena, on the other hand, may be driven more by disrupted visual input, particularly from the periphery. Determining whether increasing cholinergic tone fails to treat these misperceptions as successfully as it does complex VH would lend support to this idea.

**Figure 3:**
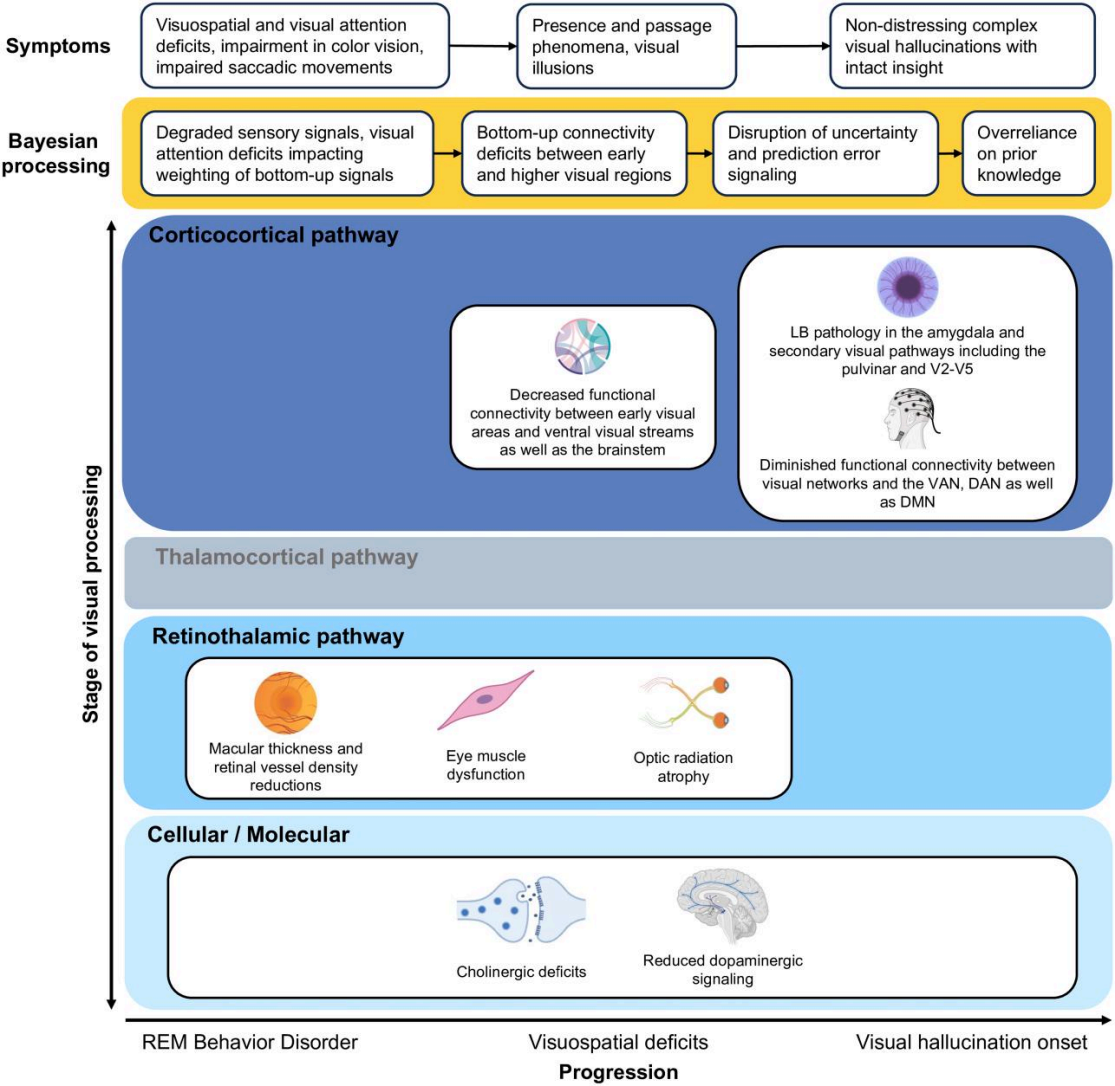
Dementia with Lewy bodies within a Bayesian framework. The x-axis depicts syndrome progression, and the y-axis illustrates successive stages of visual processing, from cellular/molecular mechanisms to retinothalamic and thalamocortical pathways, and finally corticocortical circuits. Boxes surrounded by yellow mark the aspect of Bayesian processing disrupted at each stage, with associated symptoms shown directly above. DLB is characterized by early cholinergic deficits that contribute to visuospatial and attentional impairments, as well as presence and passage phenomena and visual illusions, all of which alter the weighting of bottom-up sensory signals. These changes are compounded by dopaminergic dysregulation, disrupted functional connectivity between early visual regions and higher-order dorsal and ventral attention networks, and the accumulation of alpha-synuclein pathology in the amygdala and visual association cortices. Together, these alterations impair prediction error signalling and drive a compensatory over-reliance on prior beliefs. Created in BioRender. Vin, R. (2025) https://BioRender.com/n7i9xwr.

Consistent with a relative down-weighting of incoming sensory information in DLB, there is an emerging body of evidence for a relative overweighting of priors: patients with VH show greater improvement on a visual disambiguation task once given prior knowledge of the image compared to their non-hallucinating counterparts.^226^ Dysregulated dopaminergic and cholinergic signalling may also have the overall effect of boosting a gain-control mechanism that leads to relative increases in the precision of priors, further driving the over-weighting of top-down signalling (Fig. 3).

## Psychosis

### Prevalence and phenomenology of visual hallucinations

VH are a significant feature of psychotic illness, but prevalence rates vary considerably, from 4%^227^ to 64%^228^ among individuals diagnosed with schizophrenia. They typically occur alongside hallucinations in other sensory modalities, co-occurring with AH in 83–97% of cases, with a 10-to-12-fold increased likelihood of hallucinations in other sensory domains.^229-231^ On the other hand, the lifetime prevalence of individuals with psychosis who experience VH alone is approximately 5%.^230^ This is in contrast to DLB, where VH most often emerge in isolation. The observed interconnectedness between VH and hallucinations in other sensory modalities in psychosis suggests a shared mechanism underlying their emergence and manifestation.^231^

Most patients with psychosis have visual distortions, whereas outright visual hallucinations occur in a minority.^232^ These could be simple (e.g. flashes of light, stains, dots) or complex (e.g. figures, animals), and most patients with hallucinations report an admixture of the two.^233,234^ Unfamiliar human or humanoid figures with lifelike vividness are among the most common and distressing VH,^234-236^ reported by 55–70% of cases.^236,237^ Most patients perceive intrinsic motion in their VH, although the hallucinations remain stable with eye and head movements.^234^ Multimodal hallucinations are often accompanied by stronger conviction in their reality,^236^ although in larger studies these fused multimodal experiences appear to be rare.^238,239^ Psychotic VH are not typically experienced as silent, passive experiences; rather, they are rich, sometimes multisensory experiences that individuals perceive as interconnected.

### Comorbid visual processing deficits

Unlike the overt visual deficits in CBS and DLB, psychosis is accompanied by subtle visual alterations often difficult to detect without targeted testing. These deficits affect both early visual circuits and higher-order fronto-parietal regions responsible for integration and interpretation, suggesting a potential role for both bottom-up and top-down perceptual dysregulation in psychotic hallucinogenesis.

#### Fragmented object recognition deficits and impaired depth perception

The dorsal visual stream, involved in spatial awareness and action planning, plays a key role in constructing an initial template of visual space during perception. This allows the organism to form a preliminary hypothesis about the visual scene and facilitates efficient allocation of processing resources, which is particularly advantageous when the visual context is fragmented or ambiguous.^240^ Primary visual cortex (V1) receives visual input via two distinct pathways: the magnocellular and parvocellular systems. Both originate from the retinal ganglion cells, pass through the LGN and terminate in V1, though they remain largely distinct throughout.^241^ Given the relatively rapid transmission of information through the magnocellular system, initial visual signals from the magnocellular pathway are directed to the prefrontal cortex, where they are integrated with stored representations to generate a preliminary visual template, which is relayed as feedback to the ventral visual stream. This feedback is compared with parvocellular feedforward signals to support object recognition.^242,243^ This framing process is negatively impacted in schizophrenia; when presented with a series of progressively defragmented stimuli, patients require significantly more complete images to identify the figures compared to healthy controls.^244^ Recent work has also highlighted links between the framing process and susceptibility to experimentally induced expectation-based hallucinations.^10,245^

Binocular depth perception also relies on the coordinated integration of dorsal and ventral visual stream inputs, and disruptions in this process may contribute to impairments in psychotic illness,^246,247^ as observed in chronic and first-episode drug-naive patients^248-250^ as well as in schizotypal samples in the general population.^251^

#### Impaired global advantage

The tendency for humans to perceive the overall structure of a visual scene before focusing on finer details is termed ‘global advantage’ and has been found to be disrupted in individuals with psychotic illness.^240^ In a local-global processing task, participants were presented with visual stimuli that comprised large letters (global level) made up of smaller letters (local level).^252^ In contrast to healthy controls, patients with schizophrenia exhibited local interference effects in their reaction times for processing stimuli at the global level. In other words, they exhibited a local-biased visual processing style, prioritizing local features over global ones, unlike the global-first pattern in healthy controls.

#### Impairments in vision-related reality and source monitoring

Reality monitoring, a subdomain of source monitoring, is the cognitive process of determining whether incoming information originates from an internal or external source.^253^ This has been of particular interest in cognitive models of psychosis, as it is hypothesized by some to be a driving mechanism of hallucinations and delusions.^254^ While reality monitoring deficits are observed in individuals with psychosis more broadly,^255,256^ evidence suggests that those with VH specifically misattribute visual information.^257,258^

### Visual system pathology

Visual processing circuit abnormalities appear to be present in patients with schizophrenia, particularly in those experiencing VH. These range from cellular abnormalities in early visual structures and disruptions in molecular neurotransmitter pathways to modifications in structural and functional connectivity between visual regions.

#### Morphological and functional abnormalities of the retina

Patients with schizophrenia exhibit reduced electroretinographic a-wave and b-wave amplitudes in response to flashes of light relative to healthy controls, indicative of impaired photoreceptor function.^259^ Rod photoreceptor a-wave amplitude specifically correlates negatively with positive symptom severity and returns to normal with symptom improvement.^260,261^ Schizophrenia is also associated with abnormalities in bipolar cell function, delayed action potential output from retinal ganglion cells and disrupted centre-surround suppression.^261-263^

These functional abnormalities may be associated with retinal structural alterations in schizophrenia.^264^ Optical coherence tomography studies have reported retinal nerve fibre layer thinning^265^ and reduced macular inner ring thickness and macular volume^265,266^ in patients versus healthy controls, although these changes may reflect Wallerian and retrograde degeneration arising from disruptions further along the visual pathway, as observed in neurodegenerative diseases.^267-270^

Interestingly, several of these abnormalities are also observed in young offspring at high genetic risk for psychosis, unaffected first-degree relatives of individuals with schizophrenia, as well as those at clinical high risk for psychosis (CHR-P).^259,271,272^ This suggests that retinal structural and functional abnormalities emerge early and may serve as potential markers of the disease.

#### Impaired functioning of the magnocellular visual pathway

Recent research has also revealed impaired functioning of the downstream magnocellular visual pathway.^262,266^ The healthy magnocellular pathway is responsive to low spatial and high temporal frequencies.^241^ Psychosis-specific deficits in processing these stimulus types have been demonstrated in both fMRI and EEG settings.^273,274^ Similar findings have been observed in CHR-P individuals.^275^ The magnocellular pathway is also rich in N-methyl-D-aspartate receptors (NMDAR) and is dependent on NMDAR-mediated neurotransmission,^276^ rendering it especially sensitive to the glutamatergic dysregulation observed in schizophrenia.

#### Structural disruptions in vision-related regions

Disruption of the structural integrity of visual pathways and associated cortical regions has been implicated in psychotic disorders, particularly in those with VH. Occipital white matter integrity is reduced in the optic radiations of adults and adolescents with schizophrenia,^277-280^ and patients with childhood-onset schizophrenia exhibit significant grey matter loss and reduced cortical thickness in the left occipital cortex and temporal lobe compared to healthy controls.^281^

A similar pattern is seen in Brief Psychotic Disorder, a condition characterized by hallucinations, delusions and other psychotic symptoms but shorter in duration than schizophrenia. These individuals exhibit reduced cortical thickness in the visual and auditory association cortices compared to healthy controls.^282^ Patients with severe VH demonstrate greater reductions in left occipitotemporal cortical thickness, while those with low symptom severity show no significant deficits relative to controls.^281^ This symptom-specific finding echoes prior studies showing higher fractional anisotropy of white matter tracts connecting the hippocampus to the frontal and occipital lobes in schizophrenia patients with both VH and AH, compared to those with only AH.^283^ Collectively, these findings underscore the critical role of structural abnormalities in occipital and temporal regions, as well as disruptions in associated white matter pathways, in the pathophysiology of VH in psychotic disorders.

#### Functional disruptions in vision-related regions

VH in schizophrenia may result from impaired activation of visual brain regions and networks, as well as disrupted connectivity between these networks.^284-287^ Disrupted functional connectivity between V1 and the rest of the brain has been demonstrated during VH in psychotic disorders,^282^ with individual V1 activation correlating with VH vividness.^282^ Reduced functional involvement of V1 has also been shown through electrophysiological studies measuring visual evoked potential (VEP) abnormalities in individuals with schizophrenia.^288^ In addition, patients, but not healthy controls, exhibited an increase in amplitude of a fronto-central component of the VEP, which was inversely correlated with the reduced amplitude of the occipital component. This increase in frontal activity that accompanies the reduction in occipital activity in patients (termed ‘hyperfrontality’) has been postulated to represent additional processing that compensates for early visual deficits.^288,289^ Concurrently, higher-order visual association cortices and dorsal and ventral attention network regions have been found to exhibit increased activation, which itself positively correlates with hallucination severity.^282,284-286^ This is broadly consistent with emerging theories surrounding hallucinations as a top-down effect on perception, as feedback projections from higher processing regions are more likely to converge on visual cortical regions outside primary visual cortex.^290^

Functional connectivity aberrations in schizophrenia patients with VH extend beyond vision- and attention-related networks to include disrupted interactions between visual and limbic regions. In a resting state fMRI study, patients with VH were found to exhibit hyperconnectivity between the amygdala and the visual cortex, the left temporal pole and the inferior frontal gyrus, compared to those with only AH, or no hallucinations.^287^ The involvement of limbic structures may reflect the startling or unsettling experience of perceiving something in one’s environment that is not actually there, particularly given the widespread amygdalar involvement in DLB as well, which is characterized by predominantly non-distressing VH.

These findings, spanning multiple studies and imaging modalities, support a model of psychotic VH in which a dissociation of higher-order visual processing areas and frontal regions from V1 biases conscious perception away from sensory evidence and towards internally generated information.^286^

#### NMDA receptor hypofunction

The NMDAR hypofunction model posits a critical role for glutamate dysregulation in the pathophysiology of psychosis.^291^ Severe NMDAR hypofunction (e.g. via antagonists such as ketamine, PCP, or MK-801, or in the context of anti-NMDAR encephalitis) has been found to produce psychotomimetic symptoms in humans and animal models.^292^ Computational and theoretical studies have suggested that NMDAR hypofunction is an early insult, potentially driving compensatory hyper-precision of priors and hallucinogenesis.^6,293^ This is supported by electrophysiological evidence showing that NMDAR preferentially regulate the firing rates of inhibitory GABA interneurons, which in turn control the activity of glutamatergic pyramidal neurons. NMDAR hypofunction therefore reduces excitatory drive onto these interneurons, decreasing their firing and leading to the disinhibition of cortical pyramidal neurons, particularly in the magnocellular visual pathway in the context of VH.^6^ This cortical hyperexcitability may amplify bottom-up sensory noise, disrupt prediction error signalling and result in a compensatory overreliance on priors during perception. Recent research in animal models provides preliminary evidence for these theories, showing that globally induced NMDAR hypofunction causes a significant increase in the activation of top-down axons from the anterior cingulate cortex (ACC), accompanied by a simultaneous ACC-dependent suppression of spontaneous activity in V1.^294^ A reduction in V1 sensory-evoked activity was also found, which is consistent with the pattern of functional deviation observed in schizophrenia patients experiencing VH.

### Implications for treatment

#### Modulation of dopaminergic signalling

Classic antipsychotic medications function by blocking the D2 dopamine receptor and consistently decrease hallucination frequency and delusional conviction in roughly 70% of people experiencing psychosis.^295^ This is consistent with research showing that DA signalling regulates a gain-control mechanism, enhancing prior precision and increasing hallucinatory tendencies.^6^ This bias towards priors further strengthens with increased DA levels in schizophrenia patients and healthy controls following amphetamine administration.^13^ In another study with mice, hallucination-like percepts were found to be preceded by elevated striatal DA levels, could be induced by optogenetically stimulating mesostriatal DA neurons, and reversed by haloperidol administration.^15^

#### Modulation of cholinergic signalling

As discussed in the DLB section above, cholinergic signalling may play a key role in modulating sensory precision, biasing perceptual inference towards sensory data.^6^ Postmortem studies have found reduced expression of nicotinic and muscarinic cholinergic receptors in the hippocampus, prefrontal cortex and cingulate cortex of schizophrenia patients.^296-300^ Additionally, muscarinic receptor antagonists induce psychosis-like symptoms in healthy individuals, worsen conditioned hallucinations,^301^ and exacerbate existing symptoms in schizophrenia patients.^300^ Consistent with these findings, a variety of cholinergic treatments targeting psychotic symptoms like VH have been developed in the last two decades. AChEIs, which enhance acetylcholine signalling, may reduce prior weighting and alleviate VH severity in psychosis. Rivastigmine, in particular, has been shown to significantly reduce positive and negative symptom severity, decrease VH frequency, and alleviate associated distress,^302,303^ though these effects have yet to be confirmed in formal clinical trials, and the drug is not currently approved for the treatment of psychosis.

A more specific, targeted approach to increase acetylcholine levels that has been recently explored is the combination of muscarinic cholinergic receptor agonist xanomeline and the peripheral cholinergic receptor antagonist trospium to maximize tolerability. Having demonstrated efficacy in reducing positive psychotic symptoms in Phase II^304^ and III trials,^305^ the combination drug (brand name Cobenfy) has now become the first non-dopaminergic antipsychotic approved by the US Food and Drug Administration. Recent positive results from muscarinic M1 positive allosteric modulators suggest that Cobenfy may be the first of many cholinergic antipsychotics.^306^

### Psychosis in a Bayesian framework

**Figure 4:**
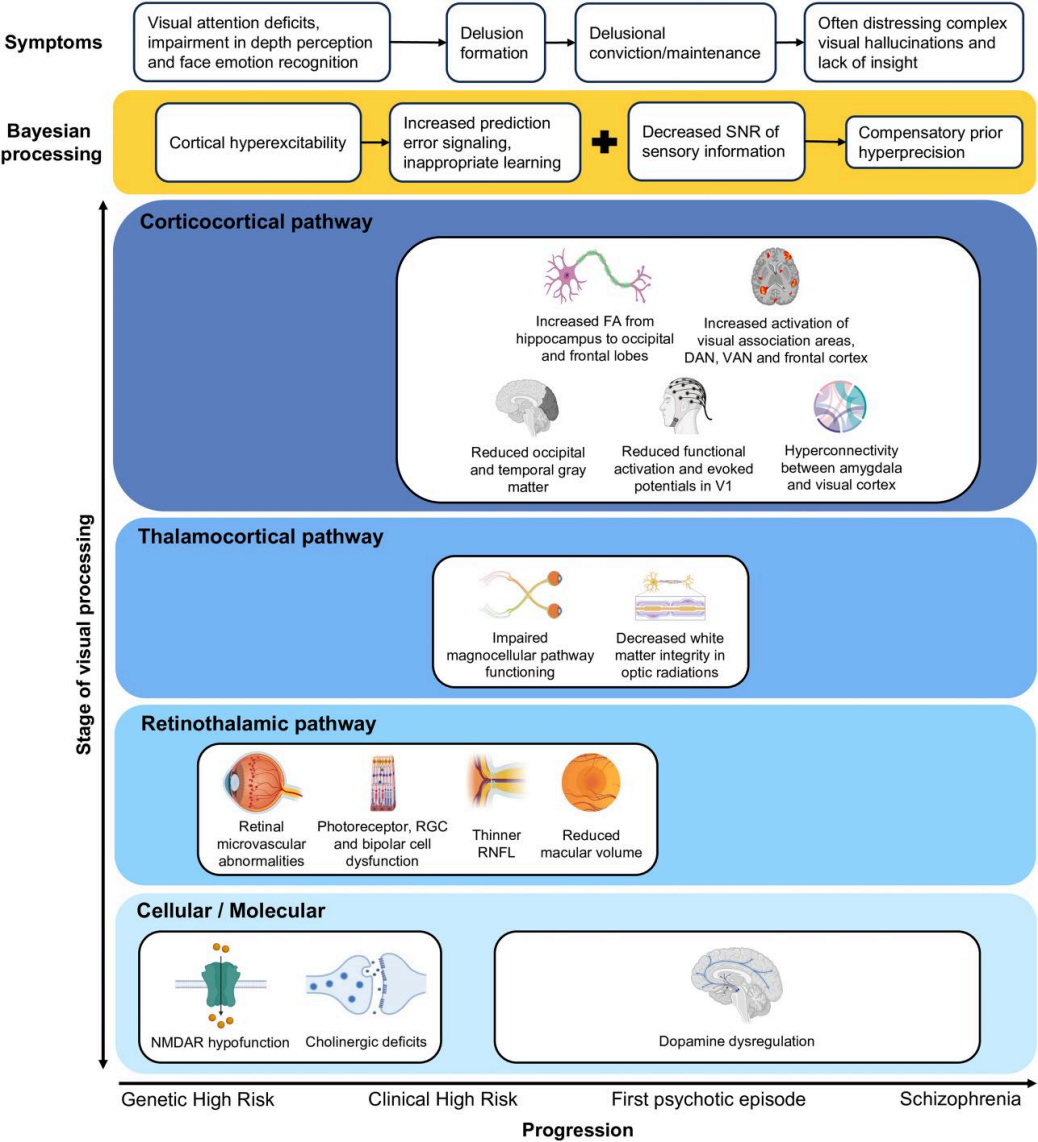
Psychosis within a Bayesian framework. The x-axis depicts syndrome progression, and the y-axis illustrates successive stages of visual processing, from cellular/molecular mechanisms to retinothalamic and thalamocortical pathways, and finally corticocortical circuits. Boxes surrounded by yellow mark the aspect of Bayesian processing disrupted at each stage, with associated symptoms shown directly above. Early stages of illness are marked by NMDAR hypofunction, resulting in cortical hyperexcitability, along with cholinergic deficits and dopaminergic dysregulation. These alterations coincide with structural and functional abnormalities in retinothalamic and thalamocortical pathways, as well as in cortical regions. The combined effects of these changes heighten prediction error signalling and promote inappropriate learning, facilitating delusion formation and ultimately driving a compensatory over-reliance on priors. SNR: signal-to-noise ratio; FA: fractional anisotropy; RGC: retinal ganglion cell; RNFL: retinal nerve fibre layer. Created in BioRender. Vin, R. (2025) https://BioRender.com/gsw0qgj.

Similar to VH in CBS and DLB, the VH of psychosis may be thought to arise from a combination of bottom-up signal disruptions and top-down compensatory mechanisms. First, VH are associated with structural abnormalities and functional disengagement of early visual structures that range from the retina (photoreceptors and retinal ganglion cells, the retinal nerve fibre layer, and the magnocellular visual pathway) all the way to V1 (grey matter loss associated with VH severity and negligible functional activation). Second, VH are associated with an increase in functional activity of visual association cortices and higher order regions in the frontal cortex, as well as increased structural integrity of white matter tracts connecting the hippocampus to these regions. These combined disruptions may be conceptualized within a similar motif as CBS and DLB, in which early visual system insults and ascending cortical noise contribute to signal disruption and a compensatory reliance on prior perceptual beliefs that drives VH emergence (Fig. 4).

While this review focuses on the emergence of VH, it is important to recognize that auditory hallucinations are more prevalent in psychosis. Previous work from our group, using a Pavlovian conditioning paradigm, has shown that auditory verbal hallucinations likewise arise from overweighted perceptual priors,^9^ with conditioned auditory hallucinations associated with heightened engagement of the auditory cortex and posterior superior temporal sulcus. This framework therefore not only accounts for both modalities but also highlights differences, suggesting that auditory and visual hallucinations may be distinguished based on differential top-down engagement of modality-specific brain regions in response to overt or subtle disruptions in bottom-up sensory input.

## Discussion

We have chosen to consider three hallucinatory disorders that vary in the directness of their aetiological connection to sensory disruption in the hopes of highlighting similarities and differences that may help us understand the mechanisms driving hallucinogenesis across disorders. This is only useful if we adopt a common framework for understanding how sensory information factors into perception. The Bayesian Brain Hypothesis suggests that the brain must build an internal model (i.e. prior beliefs) of its environment and use new sensory information to update this model. In the event of a mismatch between incoming sensory signals and the existing internal model, a prediction error signal is created, which facilitates belief updating and subsequent learning.^6^ Experiments based on this framework have demonstrated repeatedly that a propensity towards hallucinations is related to a tendency to weigh perceptual priors more heavily than incoming sensory evidence during perception.^8^ This appears to be true in a range of disorders, including psychosis,^9,10,13^ the clinical high risk state for psychosis^11,307^ and DLB,^226^ as well as patients who have no diagnosable disorder but who experience hallucinations frequently.^9,12,14^ This tendency to over-rely on priors during perception appears to vary with symptom severity,^10^ supporting the possibility that this information processing abnormality is not a static risk factor, but rather a state just proximal to symptom expression that itself may be a response to upstream causes.

We^16^ and others^308,309^ have proposed that one factor driving hallucinogenesis across disorders may be sensory disruptions: if perception is the updating of an internal model of an organism’s environment using reliability-weighted sensory information, learning that the senses provide unreliable information could tip perception in favour of expectations and drive development of hallucinations. Hallucinations arise not only when sensory information is pathologically disrupted, but also in cases where sensory information is suddenly cut off or becomes unreliable in the environment. Classic studies using sensory deprivation demonstrate that these conditions are sufficient for development of hallucinations in otherwise non-hallucination-prone individuals^310-312^ and that even brief periods of isolation can make hallucinations more likely in already hallucination-prone participants.^313,314^ Interestingly, following findings that AH in psychosis are worse in those who are socially isolated, Hoffman proposed that social deafferentation—a cutting off of social input—may play a causal or aggravating role in hallucinations via similar mechanisms.^225^

While diminished signal readily produces hallucinations, increased environmental noise also appears to be a sufficient cause. Reporting false alarms during the presentation of white noise has been repeatedly associated with hallucination proneness both in the general population and in psychotic participant samples.^9,315-318^ These observations have been classically attributed to the top-down influence of suggestion projecting upon unformed input. But even in the absence of outright suggestion, sensory noise can lead to hallucinatory phenomena. Returning to Musical Ear Syndrome, some studies have shown that the majority of patients who receive cochlear implants due to progressive deafness do not experience hallucinatory music until some (noisy) input is restored.^319^

Within our chosen Bayesian framework, then, we might conclude that it is not necessarily diminished signal but rather degradation of signal relative to noise that drives learning about the reliability of incoming sensory information and subsequent development of hallucinations. The brain has developed evolutionarily conserved strategies for dealing with low signal-to-noise ratios from sensory signals, including the use of redundant signals, neural averaging, and use of prior knowledge in the filtering of sensory information.^320^ Specifically, organisms develop estimates of the reliability of sensory information (defined as the inverse variance of the sensory signal), which are used to weight those signals in updating beliefs about the environment.^321-326^

**Figure 5:**
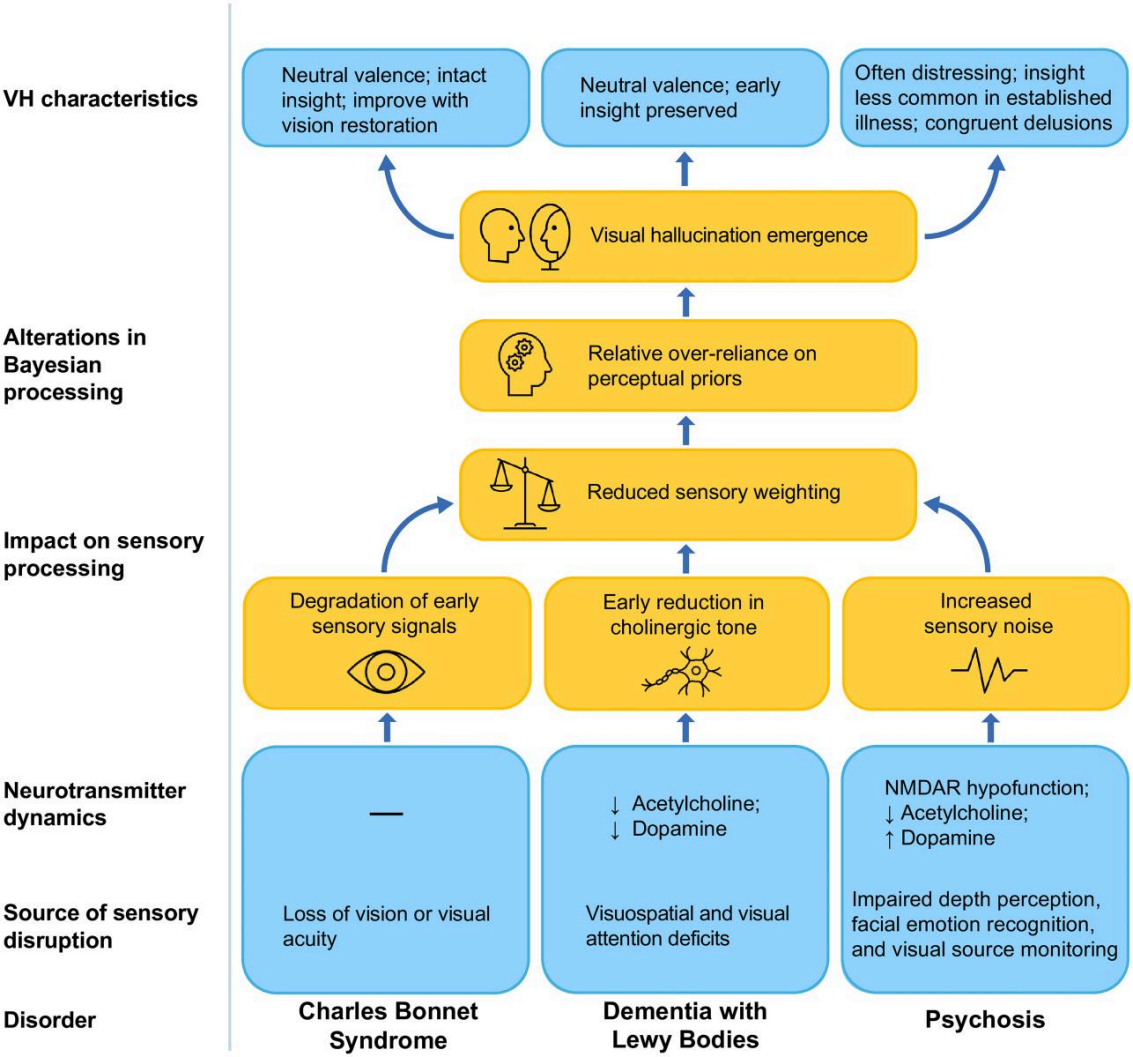
The Bayesian framework as a unifying account of hallucination emergence. CBS, DLB, and psychosis each involve distinct forms of disruption to the visual pathway and characteristic neurotransmitter alterations—most prominently in acetylcholine, dopamine and NMDAR signalling. Loss of vision in CBS, visuospatial and attention deficits in DLB and higher-level visual abnormalities coupled with NMDAR hypofunction in psychosis, all reduce the weighting of sensory information, increasing reliance on perceptual priors and facilitating the emergence of visual hallucinations. Although the underlying Bayesian mechanism is shared across the three disorders, the phenomenology of VH diverges in ways that mirror the severity and nature of the visual insult. Clear deafferentation in CBS and reduced cholinergic tone in DLB typically give rise to neutrally valenced hallucinations with relatively preserved insight, especially early in the disease course. In contrast, psychosis—characterized by less overt early visual disruption but evidence for increased sensory noise—tends to produce more distressing hallucinations, diminished insight in established illness, and co-occurring delusions. Thus, the Bayesian model provides a common mechanistic framework for hallucination emergence across these disorders, while also accounting for the disorder-specific differences shaped by the overtness of visual pathway disruption.

On examination of CBS, DLB, and psychosis, we see evidence of contributions from low signal due to deafferentation (CBS & DLB), diminished sensory precision (DLB) and enhanced noise (psychosis). Among the disorders considered here, CBS has the most direct relationship between a bottom-up insult and subsequent development of hallucinations. Phenomenological characteristics of CBS hallucinations reflect this relationship, with hallucinations almost always restricted to the visual modality (and even the impaired visual field)^52^ and occurring within hours or days of sudden vision loss.^327^ The content of VH across these disorders also varies widely and may be tied to their inciting insult. In syndromes where there is either clear deafferentation (CBS) or loss of sensory precision (DLB), hallucinations are most often neutrally valenced, if sometimes unpredictable and distracting, and those who experience them typically retain insight into their nature, especially early in the disease course. However, in psychosis, distress is common and congruent with distressing belief structures, while insight is less common in fully-formed illness (Fig. 5). This may reflect the state of uncertainty due to bottom-up noise that characterizes initial delusional belief formation. Existing in an uncertain state of the world is aversive, and resolution of that uncertainty may be a driving force behind solidification of existing beliefs and formation of hallucinations due to a compensatory over-weighting of perceptual priors.

The three disorders also highlight the importance of acetylcholine and DA in the model. The weighting of priors is governed by both sensory precision and the precision of existing beliefs; acetylcholine has been shown to modulate the former and DA the latter. In a healthy system, these neurotransmitters may function synergistically to balance prior expectations and sensory input, thereby facilitating efficient, accurate perception. Both DLB and psychosis are marked by cholinergic deficits and exhibit reduced VH rates with an increase in acetylcholine levels via AChEI administration. However, they differ in dopaminergic signalling, with elevated DA levels in psychosis and reduced DA binding in DLB. This apparent discrepancy likely reflects differences in the relative disruptions and contributions of acetylcholine and DA between the two disorders, which remains to be explored.^13,15,203,204^ Indeed, given that dopaminergic changes in DLB and PD are relatively similar despite striking differences in VH prevalence, it seems plausible that DA plays a more limited role in driving VH in alpha-synucleinopathies, with cholinergic disruption serving as the more critical factor (Fig. 5).

Despite significant advances, research that integrates longitudinal designs with computational and experimental methods is needed to more precisely characterize how the Bayesian framework accounts for the mechanisms underlying visual hallucinations (see Table 1 for a detailed summary of our hypotheses and testable predictions). A key outstanding question is how fluctuations in sensory precision and the dynamic weighting of prior beliefs interact to shape perceptual inference, and how these dynamics emerge and vary across disorders. Longitudinal research in prodromal populations—such as CHR-P individuals or those with REM sleep behaviour disorder who have not yet progressed to alpha-synucleinopathy—is rare but vital, as it could reveal the earliest detectable shifts in perceptual processing that foreshadow hallucination onset. Tracking visuospatial impairments alongside hallucinatory tendencies, including experimentally induced conditioned hallucinations, may pinpoint critical inflection points at which sensory disruption begins to bias perception towards prior-driven interpretations. A second key question concerns the temporal relationship between hallucinations and other symptoms, such as delusions, which may reveal disorder-specific trajectories and computational disruptions. Emerging evidence suggests that CHR-P individuals are more likely to develop delusions before hallucinations.^328^ We hypothesize that in such cases, increased sensory noise drives maladaptive belief formation, which over time prompts a compensatory down-weighting of incoming information and overweighting of priors, ultimately contributing to the emergence of hallucinations and crystallization of delusions (Table 1). By contrast, in DLB, where hallucinations typically precede delusions^115,117,118^—and delusions frequently manifest as reduplicative paramnesias closely tied to visual perceptual symptoms^117,118,120^—we propose that a sustained reduction in sensory precision occurs early, degrading the clarity of perceptual inputs and driving overreliance on priors. Belief precision is expected to remain relatively intact initially, with delusions arising subsequently as a secondary consequence. Hierarchical Bayesian models offer a powerful means of formalizing these mechanistic hypotheses about how perturbations such as elevated sensory noise or reduced sensory precision alter the balance between priors and sensory evidence across disorders. Predictions from these models can be evaluated using behavioural paradigms that manipulate perceptual ambiguity or degrade sensory input, coupled with EEG or fMRI to identify the stages of processing where predictive inference fails. Investigating these temporal and phenomenological differences in symptom emergence across disorders may therefore deepen insight into perceptual processes and help refine the Bayesian model.

**Table 1:** Hypotheses and testable predictions across disorders and aspects of the Bayesian model

Aspect of Bayesian Model	Disorder	Hypothesis	Prediction
Loss of sensory precision → over-reliance on priors	CBS	If hallucination onset in CBS is driven by declining visuospatial input, then the timing of hallucination onset should reflect individual differences in sensory loss.	Individuals with faster visual sensory decline should experience hallucinations earlier than those with slower decline.*
	DLB	If sensory precision is compromised from the outset in DLB, perceptual inference should become biased towards prior expectations.	Visual hallucinations should emerge later than visual attention and cholinergic deficits in prodromal states of DLB.*
	DLB	If the primary driver of VH in DLB is reduced sensory precision (ACh-modulated) rather than heightened prior precision (DA-modulated), pharmacological treatment efficacy should reflect this relative primacy.	AChEIs should be more effective than dopamine antagonists in reducing hallucinations in DLB, by directly enhancing sensory precision without risking motor side effects.*
	Psychosis	If increased sensory noise drives inappropriate belief-updating and delusion formation, and hyper-precise priors emerge as a compensatory response, the temporal order of symptom onset should reflect this progression.	Hallucinations should emerge later than delusions in a significant proportion of those with psychosis.^328^
	Psychosis	If psychosis subgroups differ in initial excitability profiles, the temporal sequence of delusion versus hallucination onset should diverge across subgroups.	A significant subset of individuals with psychosis with greater baseline cortical excitability should show delusions preceding hallucinations, while another subset with lower cortical excitability might show a different pattern.*
	DLB/CBS versus Psychosis	If psychosis is characterized by early cortical hyperexcitability that destabilizes inference and gradually degrades sensory precision, this should be reflected in the timing of hallucination onset relative to conditions where sensory precision is compromised from the outset, such as DLB and CBS.	Hallucinations should appear earlier in the disease course of DLB and CBS, closely linked to sensory deficits, but later in psychosis after progressive sensory degradation.*
Hyper-precise priors → hallucinations	CBS	If hyper-precise priors compensate for deteriorating sensory precision, individuals with strongly-weighted visual priors should be more prone to perceptual ‘filling-in’ when retinal or visual pathway damage occurs.	Individuals with a stronger reliance on priors before sensory decline should be more susceptible to CBS in the face of vision loss.*
	CBS	If hallucinations in CBS stem from hyper-precise priors compensating for the loss of sensory input, then reducing prior precision should lessen hallucination severity and frequency.	Dopaminergic interventions that directly lower prior precision should be more effective for CBS than cholinergic therapies.*
	Psychosis	If hyper-precise priors increase susceptibility to hallucinations, then differences in baseline prior precision should influence the course of symptom development in psychosis.	While both baseline and compensatory prior hyper-precision may give rise to hallucinations, individuals with hyper-precise priors at baseline should present with fewer comorbidities (as sensory noise is not as prominent) and potentially lower delusion severity.*
	Psychosis	If hyper-precise priors underlie hallucinations, the relative efficacy of cholinergic versus dopaminergic therapies should differ accordingly.	Cholinergic therapies may transiently reduce hallucinations by enhancing sensory precision, but prior precision could quickly readjust, limiting sustained benefit; dopaminergic interventions, which directly reduce pathological prior weighting, are expected to provide more durable symptom relief and also restore the uncertainty present in early (prodromal) illness.*

Predictions with empirical evidence include citations; those without are marked with an asterisk (*).

A complementary strategy is to test causal mechanisms directly through pharmacological manipulations of neurotransmitter systems central to precision-weighting—notably, the cholinergic and dopaminergic pathways. These interventions could clarify the neurochemical basis of disrupted perceptual inference and determine whether recalibrating these processes can prevent or ameliorate hallucinations. Recruiting prodromal cohorts for trials testing these interventions is critical, as early-stage participants offer a unique opportunity to probe the mechanisms underlying hallucination emergence and to assess treatment efficacy before the onset of cognitive decline or other debilitating symptoms (see^329,330^ for details of such a study for CHR-P). We hypothesize that although both cholinergic and dopaminergic medications have the potential to rebalance the ratio between the precision of sensory evidence and the precision of priors, their long-term pharmacological and therapeutic effects will depend on the specific computational mechanisms underlying each disorder. In conditions such as DLB, dopamine antagonists may reduce hallucinations by lowering prior precision, but at the cost of exacerbating motor symptoms, and in any case elevated prior precision is not the primary deficit in DLB. In this context, cholinergic agents may be relatively more beneficial, as they directly target the underlying deficit in sensory precision. Conversely, in disorders such as psychosis, cholinergic interventions may transiently improve hallucinations by enhancing sensory precision; however, over time, prior precision may readjust or even strengthen to compensate, potentially leading to relapse with more severe hallucinatory symptoms. In such cases, dopaminergic modulation may therefore prove more advantageous, as it directly attenuates the pathological overweighting of priors (Table 1). Computational modelling approaches that estimate patient-specific sensory and prior precision could predict individual treatment responsiveness, paving the way for stratified and precision interventions.

Together, these integrated approaches could transform the Bayesian account from a descriptive framework into a predictive, experimentally grounded model that can guide early and targeted intervention strategies.

## Data Availability

Data sharing is not applicable to this article as no new data were created or analysed in this study.
